# The management strategies of cancer-associated anorexia: a critical appraisal of systematic reviews

**DOI:** 10.1186/s12906-018-2304-8

**Published:** 2018-08-09

**Authors:** Fangyuan Zhang, Aomei Shen, Yinghui Jin, Wanmin Qiang

**Affiliations:** 1 Tianjin Medical University Cancer Institute and Hospital, National Clinical Research Center for Cancer; Key Laboratory of Cancer Prevention and Therapy, Tianjin; Tianjin’s Clinical Research Center for Cancer, Tianjin, CN, Hexi District, 1 West Lake Road, Tianjin, China; 2 Center for Evidence-Based and Translational Medicine, Zhongnan Hospital of Wuhan University, Wuhan, CN, Center for Evidence-Based and Translational Medicine, Wuhan University, Wuhan, China

**Keywords:** Evidence-based medicine, Cancer, Anorexia, Systematic review, Meta-analysis

## Abstract

**Background:**

Cancer-related anorexia remains one of the most prevalent and troublesome clinical problems experienced by patients with cancer during and after therapy. To ensure high-quality care, systematic reviews (SRs) are seen as the best guide. Considering the methodology quality of SRs varies, we undertook a comprehensive overview, and critical appraisal of pertinent SRs.

**Methods:**

Eight databases (between the inception of each database and September 1, 2017) were searched for SRs on the management of cancer-related anorexia. Two researchers evaluated the methodological quality of each SR by using the Revised Assessment of Multiple Systematic Reviews (R-AMSTAR) checklist. Characteristics of the “high quality” SRs were abstracted, included information on relevant studies numbers, study design, population, intervention, control, outcome and result.

**Results:**

Eighteen SRs met the inclusion criteria. The R-AMSTAR scores of methodological quality ranged from 18 to 41 out of 44, with an average score of 30. Totally eight SRs scored ≥31 points, which showed high methodological quality, and would be used for data extraction to make summaries. Anamorelin had some positive effects to relieve cancer anorexia-cachexia syndrome (CACS) and improve the quality of life (QoL). Megestrol Acetate (MA) could improve appetite, and was associated with slight weight gain for CACS. Oral nutritional interventions were effective in increasing nutritional intake and improving some aspects of QoL in patients with cancer who were malnourished or at nutritional risk. The use of thalidomide, Eicosapentaenoic Acid, and minerals, vitamins, proteins, or other supplements for the treatment of cachexia in cancer were uncertain, and there was inadequate evidence to recommend it to clinical practices, the same situation in Chinese Herb Medicine and acupuncture (acupuncture and related therapies were effective in improving QoL) for treating anorexia in cancer patients, warranting further RCTs in these areas.

**Conclusions:**

Anamorelin, MA, oral nutrition interventions, and acupuncture could be considered to be applied in patients with cancer-related anorexia. Future RCTs and SRs with high quality on the pharmaceutical or non-pharmaceutical interventions of anorexia in cancer patients are warranted.

**Electronic supplementary material:**

The online version of this article (10.1186/s12906-018-2304-8) contains supplementary material, which is available to authorized users.

## Background

Cancer is the second leading cause of death worldwide, Global Burden of Disease Cancer Collaboration reported that there were 17.5 million cancer cases around the world and 8.7 million deaths in 2015; cancer cases increased by 33% during the last decade [1]. Towards the end of life, individuals with cancer experience substantial symptom burden [2, 3]. The top three common symptoms in patients with cancer at the end of life are fatigue, pain, and anorexia (appetite loss) [4, 5]. Anorexia is defined as loss of appetite with or without weight loss, which occurs in half of newly diagnosed cancer patients and 26.8%~ 57.9% of patients with advanced cancer [3, 6]. Cancer-related anorexia is a major clinical problem, and adversely influences nutritional status of patients, which may negatively impact patients’ quality of life and increase the burden on healthcare resources [7]. It is also upsetting to both patients and their caregivers, who need supportive care from healthcare professionals [8]. Besides, anorexia is one of the independent prognostic factors for survival [9]. Thus, scientific and effective management strategies for cancer-related anorexia are urgently needed.

Systematic reviews (SRs) provide an opportunity to base decisions on accurate, succinct, credible, and comprehensive summaries of the best available evidence on a specific topic, and act as one of the key tools for healthcare professionals to achieve evidence based decisions [10]. To date, several SRs on the management of anorexia in cancer patients have been published. Considering the methodology quality of SRs varies, and uncritically accepting the results of a systematic review has a risk. Therefore, we sought to conduct a comprehensive overview, and critical appraisal of pertinent SRs to better characterize the management strategies of cancer-related anorexia, based on SRs with high methodological quality.

## Methods

### Identification of studies

The following electronic databases were systematically searched for SRs: PubMed, Embase, The Cochrane library, CINAHL, JBI, China National Knowledge Infrastructure (CNKI), Chinese Bio-medical Literature Database (CBM), and WanFang Database. Articles published in English and Chinese between the inception of each database and September 1, 2017, were searched for controlled vocabulary terms specific to each database related to neoplasms, anorexia, systematic reviews, meta analysis. Detailed search strategies were provided in Additional file 1. The references of SRs included were also manually reviewed.

### Eligibility criteria

We defined the following inclusion criteria: (1) Population: Adults with cancer (all sites and stages) suffering from anorexia or symptoms indicative of anorexia, such as lack of appetite, weight loss, poor performance status, and diminished quality of life [6]; (2) Interventions: Pharmaceutical or non-pharmaceutical treatments, such as exercises, oral nutrition intervention, acupuncture, in cancer patients with anorexia; (3) Included studies: At least 2 studies, either randomized clinical trials (RCTs) or observational studies; (4) Design: Systematic review/meta-analysis in accordance with Cochrane Collaboration; and (5) Form: Full texts available. Exclusion criteria included: (1) Population: Animals, children with cancer, or non-cancer patients. (2) Interventions: Qualitative researches on experience or psychosocial effect of anorexia in cancer patients; (3) Design: Protocol, overview of SRs, narrative review, expert review, scoping review.

### Selection of studies

Two researchers (FY, AM) assessed the eligibility of all SRs independently. For the process, titles and abstracts were reviewed firstly; then full-text articles were reviewed to judge the eligibility. Full texts that did not fulfill the priori defined inclusion criteria were excluded. Disagreements regarding inclusion in the final review were resolved through discussion and consensus. A third researcher (YH) was consulted if the disagreements cannot be resolved between the two researchers.

### Methodological quality of studies and data extraction

Two researchers (FY, AM) evaluated the methodological quality of each SR by using the Revised Assessment of Multiple Systematic Reviews (R-AMSTAR) checklist, a tool with 11 items [11]. The R-AMSTAR items were scored with a range of one to four points with higher scores indicating better methodological quality. The total score on R-AMSTAR was 44 points, and the minimum score is 11points. Any disagreements between the researchers regarding to the methodological quality of included SRs were resolved in a consensus-building discussion, if necessary by a third researcher (YH). Besides, only those with the R-AMSTAR score ≥ 31 points, defined as “high quality”, were taken into consideration [12, 13].

Characteristics of the included “high quality” SRs were abstracted independently by two researchers (FY, AM), and disagreements were resolved through discussion and consensus. Abstracted data was included information on relevant studies numbers, study design, population, intervention, control, and outcome. As for SRs with the same intervention, the latest one with better methodological quality would be included for analysis.

## Results

### Study selection

**Fig. 1:**
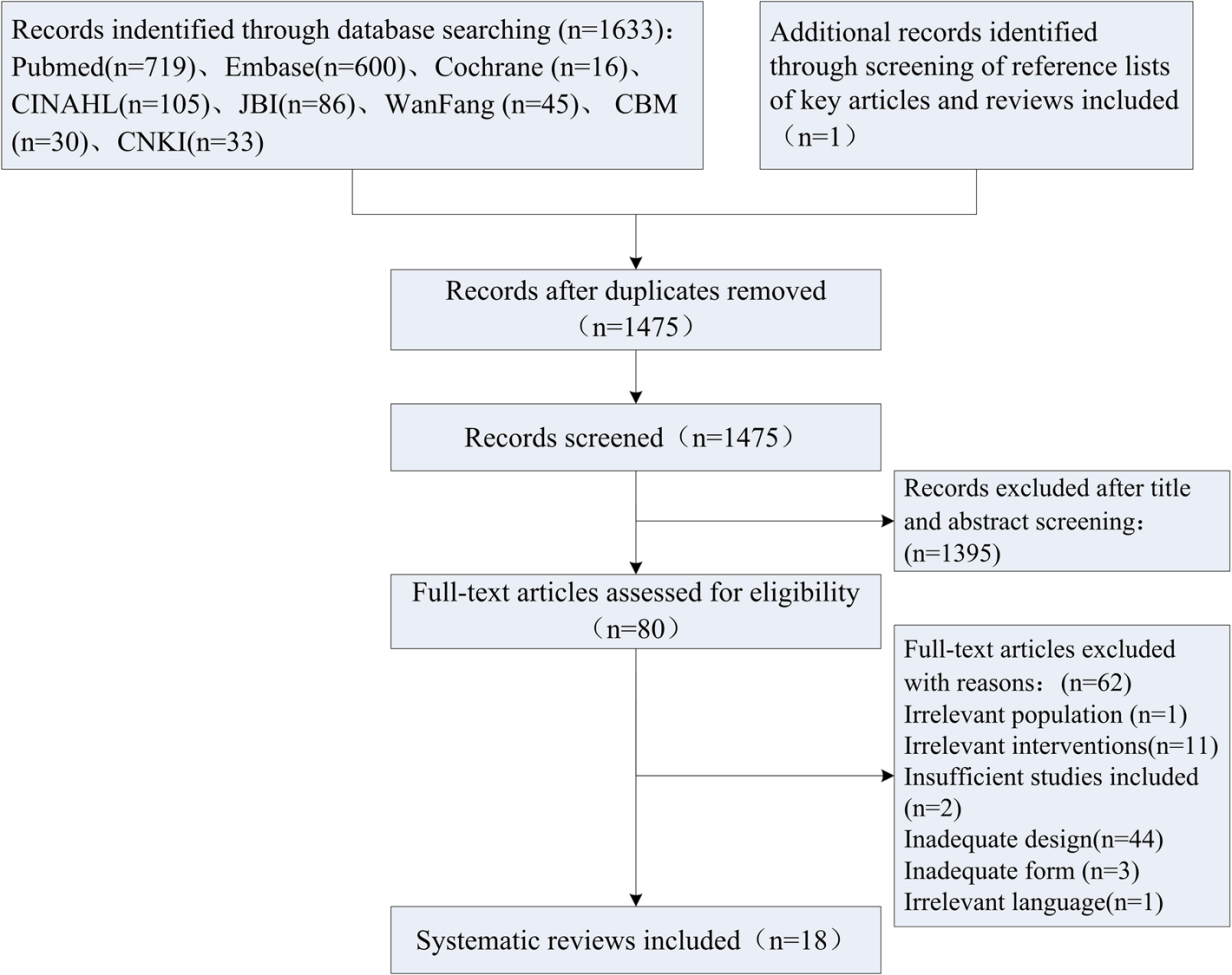
Flowchart of the systematic review selection procedure

The review identification and selection procedures are outlined in Fig. 1. Of 1634 initially identified search hits, 159 duplications were excluded, and 1475 records remained for citation screening. After the screening of titles and abstracts, 80 full texts were retrieved for eligibility assessment. Among them, 62 publications were excluded because of the following reasons: irrelevant population (*n* = 1), irrelevant interventions (*n* = 11), insufficient studies included (*n* = 2), inadequate design (*n* = 44), inadequate form (*n* = 3), irrelevant language (*n* = 1). As a result, 18 SRs met the predefined eligibility criteria were included [6, 14–30].

### Methodological quality

Results of the critical appraisal for 18 SRs using R-AMSTAR are presented in Table 1. The R-AMSTAR scores of methodological quality ranged from 18 to 41 out of 44, with an average score of 30. Totally 8 SRs (Dewey et al., 2007; Baldwin et al., 2012; Reid et al., 2012; Ruiz et al., 2013; Chung et al., 2016; Lau et al., 2016; Mochamat et al., 2017; Bai et al., 2017) [17, 19–21, 26–29] scored ≥31 points, which showed high methodological quality, and would be used for data extraction to make a summary. Most SRs provided “a priori” design, i.e., a protocol, a research question and inclusion criteria (mean: 3.33). In contrast, the criteria of items such as “quality of included studies assessed and documented” (mean: 2.06), “scientific quality used appropriately in formulating conclusions” (mean: 1.22), and “conflict of interest stated” (mean: 2.33) were completely satisfied only by one SR. The items of “list of studies included and excluded”, and “likelihood of publication bias assessed” were also insufficient in several reviews.

**Table 1:** Assessment of the methodological quality using R-AMSTAR

Study	R-AMSTAR items											Score
	1. “A priori” design provided?	2. Duplicate study selection and data extraction?	3. Comprehen-sive literature search?	4. Status of publication as an inclusion criterion?	5. List of studies (included and excluded)?	6. Characteris-tics of the included studies?	7. Quality of included studies assessed and documented?	8. Scientific quality used appropriately in formulating conclusions?	9. Appropriate methods used to combine the findings of studies?	10. Likelihood of publication bias assessed?	11. Conflict of interest stated?	
Maltoni et al., 2001 [14]	4	4	4	2	1	4	1	1	1	1	1	24
Pascual ea. al,2004 [15]	3	3	3	2	1	4	2	1	4	1	2	26
Yavuzsen et al., 2005 [6]	3	2	1	1	1	4	1	1	1	1	2	18
Berenstein & Ortiz, 2005 [16]	3	4	4	4	1	4	2	1	4	1	1	29
Dewey et al., 2007 [17]	4	4	4	4	3	4	2	1	4	1	3	34^a^
Lesniak et al., 2008 [18]	3	3	4	2	1	4	3	1	4	2	2	29
Baldwin et al., 2012 [19]	3	4	3	1	2	4	2	1	4	4	4	32 ^a^
Reid et al., 2012 [20]	4	4	4	3	4	4	2	1	4	4	2	36 ^a^
Ruiz et al., 2013 [21]	4	4	4	4	4	4	4	2	4	4	3	41 ^a^
Payne et al., 2013 [22]	3	4	4	1	3	4	2	1	2	1	3	28
Reid et al., 2013 [23]	3	4	1	2	3	4	2	1	3	2	3	28
Solheim et al., 2013 [24]	3	4	2	2	3	4	2	1	3	1	2	27
Miller et al., 2014 [25]	3	4	1	1	2	4	2	1	2	1	2	23
Chung et al., 2016 [26]	4	2	3	4	3	4	2	1	4	4	3	34 ^a^
Lau et al., 2016 [27]	4	3	3	4	3	4	2	1	4	4	3	35 ^a^
Bai et al., 2017 [28]	3	3	3	2	3	4	2	1	4	4	3	32 ^a^
Mochamat et al., 2017 [29]	3	4	2	4	2	4	2	4	3	1	2	31 ^a^
Li et al., 2017 [30]	3	3	2	4	3	1	2	1	4	1	1	25
Mean	3.33	3.50	2.89	2.61	2.39	3.83	2.06	1.22	3.28	2.11	2.33	29.56
SD	0.49	0.71	1.13	1.24	1.04	0.71	0.64	0.73	1.07	1.41	0.84	5.47

^a^R-AMSTAR score ≥ 31 points means a high methodological quality

### Summaries of SRs with high methodological quality

Table 2 describes the general characteristics of 8 included SRs with high methodological quality. The management strategies of cancer related anorexia included drugs (*n* = 3) [20, 21, 28], dietary or nutritional intervention (*n* = 3) [17, 19, 29], Chinese Herbal Medicine (CHM, *n* = 1) [26], acupuncture (*n* = 1) [27].

**Table 2:** Characteristics of systematic reviews

Systematic review	Relevant studies, No	Study design (No)	Population	Intervention	Control	Outcome
Dewey et al., 2007 [17]	5	RCT (*n* = 5)	Incurable or advanced cancer patients with either a reported weight loss of 5% and above or cachexia	Oral fish oil supplementation	Placebo/ active matched control	AE, BC, CR, EE, Fatigue, FS, NS, PS, QoL, SA, Survival, WC
Baldwin et al., 2012 [19]	13	RCT (*n* = 13)	Adults cancer patients with malnourished or at risk of malnutrition	Dietary advice, oral nutritional supplements, or both	Usual care	NI (ie, weight loss and energy intake), QOL, Survival
Reid et al., 2012 [20]	3	RCT (*n* = 3)	Advanced or incurable cancer patients with weight loss or cachexia	Thalidomide orally	Placebo/ an alternative experimental treatment modality	AE, BC, Fatigue, FS, GP, PIC, PS, QoL, Survival
Ruiz et al., 2013 [21]	35	RCT (*n* = 35)	Patients with cancer, AIDS or another underlying pathology related anorexia-cachexia	Megestrol acetate	Placebo/ other active drug treatments/ different doses	AC, AE, MAC, QoL, TSFT, WC
Chung et al., 2016 [26]	14	RCT (*n* = 14)	Cancer patients with various types, most in moderate to advanced stage	CHM, either in combination with other treatments or used alone	Conventional treatment, placebo, or no treatment.	Fatigue, paresthesias, dysesthesias, chronic pain, anorexia, insomnia, limbs edema, constipation
Lau et al., 2016 [27]	13	RCT (*n* = 13)	Patients with various types of cancer, near half in moderate to advanced stages	Any form of acupuncture and/or related therapies	Any type of interventions without acupuncture or related treatments	Fatigue, paresthesia, dysesthesias, chronic pain, anorexia, insomnia, limb edema, constipation, QoL
Bai et al., 2017 [28]	4	RCT (*N* = 3) Randomized crossover Trial (*n* = 1)	Cancer anorexia-cachexia syndrome (CACS) patients	Anamorelin	Placebo or Anamorelin at various doses	AC, GS, LBM, PS, QoL, Serum biomarkers, WC
Mochamat et al., 2017 [29]	21	RCT (*n* = 17) Prospective studies (*n* = 3) Crossover study (*n* = 1)	Cancer patients with cachexia or cachexia-related symptoms	Vitamin, mineral, proteins, or other dietary supplements	No supplements/ different supplements	AE, AC, LBM, L-carnitine, QoL, Survival, WC,

*Abbreviations*: *AC* appetite change, *AE* adverse events, *AIDS* acquired immune deficiency syndrome, *BC* body composition, *CHM* chinese herbal medicine, *CR* compliance rates, *EE* energy expenditure, *FS* functional status, *GS* grip strength, *LBM* lean body mass, *MAC* mid-arm circumference, *NS* nutritional status, *NI* nutritional indices, *PIC* pro-inflammatory cytokines, *PS* performance status, *QoL* quality of life, *RCT* randomized clinical trial, *SE* side effects, *TSFT* triceps skin fold thickness, *WC* weight change

### Acupuncture

Compared with conventional interventions, acupuncture and related therapies improved quality of life in patients with gastrointestinal cancer (*n* = 111, pooled SMD: 0.75, 95% CI: 0.36~ 1.13); Acupuncture and related therapies also showed improvement in anorexia, but there was no statistical significance (*n* = 50, RR: 2.51, 95%CI: 0.94~ 6.72); Besides, acupuncture and related therapies significantly reduced pain (*n* = 175, pooled WMD: -0.76, 95% CI: -0.14~ − 0.39) in patients with liver or gastric cancer, and fatigue (*n* = 57, MD: -0.63, 95% CI: -1.22~ − 0.44) in lung cancer patients; Adverse events of acupuncture and related therapies were infrequent and mild [27].

### Chinese herbal medicine

Qi-ge-kai-wei decoction plus megestrol acetate versus megestrol acetate alone, showed a higher proportion of reported improvement (93.8% vs 87.5%) for treating anorexia in advanced lung cancer patients; However, there was no statistical significance. Tong-tai decoction and chemotherapy showed more improvement than chemotherapy alone in advanced colorectal cancer patients (55.0% vs 45.0%), but again no significance difference was found; CHM could significantly reduced pain (pooled WMD: -0.90, 95% CI: -1.69~ − 0.11), compared with conventional intervention; Adverse events were infrequent and mild [26].

### Eicosapentaenoic acid

There was no sufficient data to establish whether oral Eicosapentaenoic acid (EPA) was better than placebo. Comparisons of EPA combined with a protein energy supplementation versus a protein energy supplementation (without EPA) in the presence of an appetite stimulant (Megestrol Acetate) provided no evidence that EPA could improve symptoms associated with the cachexia syndrome often seen in patients with advanced cancer [17].

### Oral nutritional intervention

Oral Nutritional intervention (ONI) was associated with statistically significant improvements in weight (MD = 1.86 kg, 95% CI = 0.25 ~ 3.47), and energy intake (MD = 432 kcal/d, 95% CI = 172~ 693), compared with routine care; However, after removing the main sources of heterogeneity, there was no statistically significant difference in weight gain or energy intake; In addition, ONI had a beneficial effect on some aspects of QoL (emotional functioning, dyspnea, loss of appetite, and global QoL), but had no effect on mortality (RR = 1.06, 95% CI = 0.92 ~ 1.22) [19].

### Vitamins, minerals, proteins, and other supplements

As far as vitamins were concerned, vitamin E in combination with omega-3 fatty acids displayed a significant prolonged survival (no exact number presented, *P* = 0.01) in one RCT, vitamin D showed improvement of muscle weakness (37%) in a crossover study, and vitamin C supplementation led to significantly higher scores of various quality of life aspects, such as physical, emotional and cognitive, in a sample with a variety of cancer diagnoses; Regarding minerals, only one study examined the use of magnesium with no effect on weight loss; For proteins, a combination therapy of β-hydroxy-β-methylbutyrate (HMB), arginine, and glutamine showed an increase in body weight (2.27 ± 1.17 kg vs 0.27 ± 1.39, *P* = 0.06) after 24 weeks in a study of advanced solid tumour patients, whereas the same combination did not show a benefit on lean body mass (LBM) in a large sample of advanced lung and other cancer patients after 8 weeks; L-carnitine led to an increase of body mass index (3.4 ± 1.4% vs 1.5 ± 1.4%, *P* < 0.05) and an increase in overall survival (median 519 ± 50d vs 399 ± 43d, *P* = ns) in advanced pancreatic cancer patients; Adverse effects of food supplementation were rare and showed mild intensity [29].

### Anamorelin

Compared with placebo, Anamorelin showed statistically significant improvement in LBM (SMD = 0.34, 95% CI = 0.21~ 0.46), body weight (SMD = 1.91, 95% CI = 0.53~ 3.29), Anderson Symptom Assessment Scale (ASAS) score (MD = 8.05, 95% CI = 5.97~ 10.12), insulin-like growth factor-1 level (SMD = 2.51, 95% CI = 0.37~ 4.46), IGF binding protein-3 (SMD = 1.65, 95% CI = 1.13~ 2.18). Three studies reported non-dominant handgrip strength, but there was no significant difference (SMD = 0.30, 95% CI = − 0.12~ 0.72). All the included studies reported adverse events, Anamorelin induced fewer adverse events, but there was no significant difference between the two groups (RR = 0.07, *P* = 0.35) [28].

### Megestrol acetate

Megestrol acetae (MA) showed a benefit compared with placebo, particularly with regard to appetite improvement (RR = 2.57, 95% CI = 1.41~ 3.40) and weight gain (RR = 1.55, 95% CI = 1.06~ 2.26) in cancer, but lack of benefit when compared to other drugs. There was insufficient information to define the optimal dose of MA, but higher doses were more related to weight improvement than lower doses; Quality of life improvement in patients was observed only when comparing MA versus placebo (RR = 1.91, 95% CI = 1.02~ 3.59) but not other drugs in cancer. Oedema, thromboembolic phenomena and deaths were more frequent in patients treated with MA [21].

### Thalidomide

A dearth of large, well conducted trials about thalidomide for the management of cancer cachexia. At present, there was insufficient evidence to refute or support the use of thalidomide for the management of cachexia in advanced cancer patients [20].

## Discussions

### Quality of evidence

In general, the methodological quality of the SRs included was moderate with an average R-AMSTAR score of 30 out of 44, and had great room of improvement. Totally the average scores of 5 items of R-AMSTAR were less than 2.4, item 5 “list of studies (included and excluded)?”, item 7 “quality of included studies assessed and documented?”, item 8 “scientific quality used appropriately in formulating conclusions?”, item 10 “likelihood of publication bias assessed?”, item 11 “conflict of interest stated?”. As for item 5, most of included SRs failed to meet the criteria of “table/list/figure of excluded studies”, and “reader is able to retrace the excluded studies”, which needed to pay more attention to; About item 7 and item 8, most failed to satisfy criteria of “discussion/recognition/ awareness of level of evidence”, “quality of evidence should be rated/ranked based on characterized instruments, e.g. Grading of Recommendations Assessment, Development, Evaluation (GRADE)”, “the results of the methodological rigor and scientific quality are explicitly stated in formulation recommendations”, “have conclusions integrated/drives towards a clinical consensus statement”, and “this clinical consensus statement drives toward revision of confirmation of clinical practice guidelines”; Criteria of “an assessment of publication bias should include graphical aids” and “statistical tests” was not performed by most of included SRs, regarding to item 10; Concerning item 11, most SRs authors did not report “conflict of interest”, and “statement of support or conflict of interest in the primary inclusion studies”.

### Summaries of main findings

In regard to the management of cancer related anorexia, summaries of 8 SRs with high methodological quality include drugs, dietary or nutritional intervention, CHM, and acupuncture. Anamorelin had some positive effects to relieve cancer anorexia-cachexia syndrome (CACS) and improved QoL; However, the heterogeneity was apparent, so the clinical effects of Anamorelin should be further validated by increasing the sample size, varying the range of doses during treatment, and observing other outcomes. Megestrol Acetate (MA) could improve appetite, and was associated with slight weight gain for CACS; Despite of the fact that these patients were receiving palliative care they should be informed of the risks involved in taking MA. Oral nutritional interventions were effective in increasing nutritional intake and improving some aspects of QoL in patients with cancer who were malnourished or at nutritional risk, but did not appear to improve mortality. The use of thalidomide, EPA, and minerals, vitamins, proteins, or other supplements for the treatment of cachexia in cancer was uncertain, and there was inadequate evidence to recommend it for clinical practices, the same situation in CHM and acupuncture for treating anorexia in cancer patients, warranting further RCTs in these areas; Acupuncture and related therapies were effective in improving quality of life when compared with conventional intervention alone among cancer patients; Limitations on current evidence body imply that they should be used as complements, rather than alternatives, to conventional care.

### Strengths and limitations

Despite the care with which they were conducted, SRs might differ in quality. And yield different answers to the same question [31]. Therefore, users of SRs should be critical and careful at the methodological quality of the available reviews [32]. Thus, only SRs on management of cancer-related anorexia with high methodological quality would be taken into consideration, in order to improve the reliability of results. However, this strategy might hinder us from the chance to get access to RCTs with high quality in SRs not included.

As for the instrument to assess the quality of conduct of systematic reviews of randomised controlled trials of interventions, A Measurement Tool to Assess systematic Reviews (AMSTAR), published in 2007, was one of the most widely used instruments [33, 34]. Researchers had pointed out that AMSTAR had some limitations, and needed to be improved [35, 36]. While R-AMSTAR provided a numerical score of methodological quality for each review, and also met some limitations, e.g. criteria of discussion/recognition/ awareness of the quality of the body of evidence, based on AMSTAR [11]. R-AMSTAR was of more usable and congruent to assess methodological quality of SRs, compared with AMSTAR.

## Conclusions

Our findings of management strategies for patients with cancer-related anorexia suggested that anamorelin, MA, oral nutrition interventions, and acupuncture could be considered to be applied in clinical practices. Future RCTs and SRs with high quality on the pharmaceutical or non-pharmaceutical interventions of anorexia in cancer patients are warranted.

## Additional file

Additional file 1: Search method. (DOCX 14 kb)

## Abbreviations

AC: Appetite change
AE: Adverse events
AIDS: Acquired immune deficiency syndrome
BC: Aody composition
CACS: Cancer anorexia-cachexia syndrome
CHM: Chinese Herbal Medicine
CR: Compliance rates
EE: Energy expenditure
EPA: Eicosapentaenoic acid
FS: Functional status
GS: Grip strength
LBM: Lean body mass
MA: Megestrol acetae
MAC: Mid-arm circumference
NI: Nutritional indices
NS: Nutritional status
ONI: Oral nutritional intervention
PIC: Pro-inflammatory cytokines
PS: Performance status
QoL: Quality of life
R-AMSATR: Revised Assessment of Multiple Systematic Reviews
RCT: Randomized clinical trial
SE: Side effects
SRs: Systematic reviews
TSFT: Triceps skin fold thickness
WC: Weight change
