# Supplementary material for: The management strategies of cancer-associated anorexia: a critical appraisal of systematic reviews
Source: BMC Complement Altern Med. 2018 Aug 9;18:236. doi: 10.1186/s12906-018-2304-8 (PMC6085669; doi:10.1186/s12906-018-2304-8)
Supplement: Supplementary file 1 — Search method. (DOCX 14 kb) [file 12906_2018_2304_MOESM1_ESM.docx]

**Additional file 1:Search method**

| **Database** | **Search strategy** |
| --- | --- |
| Pubmed | #1 neoplasms[mesh]  #2 anorexia[mesh]  #3 review[publication type]  #4 meta-analysis[publication type]  #5 neoplasia*[title/abstract] or tumor*[title/abstract] or malignanc*[title/abstract] or cancer[title/abstract]  #6 anorexia[title/abstract] or cachexia[title] or anorexia-cachexia syndrome[title]  #7 systematic review*[title/abstract]  #8 #1 or #5  #9 #2 or #6  #10 #3 or #4 or #7  #11 #8 and #9 and #10 |
| Embase | #1 malignant neoplasm[emtree]  #2 anorexia[emtree]  #3 systematic review[emtree]  #4 meta analysis[emtree]  #5 cancer*[title/abstract] or malignant neoplasm*[title/abstract] or malignant neoplastic disease* [title/abstract] or malignant tumor[title/abstract] or malignant tumour[title/abstract] or neoplasia, malignant[keyword] or tumor, malignant[keyword] or tumour, malignant[keyword]  #6 anorexic[title/abstract] or anorexia[title/abstract] or cachexia[title] or anorexia cachexia syndrome[title]  #7 review, systematic[keyword] or analysis, meta[keyword] or meta-analysis[title/keyword] or metaanalysis [title/keyword]  #8 #1 or #5  #9 #2 or #6  #10 #3 or #4 or #7  #11 #8 and #9 and #10 |
| Cochrane | #1 neoplasms[mesh]  #2 anorexia[mesh]  #3 neoplasia*[title/abstract/keyword] or tumor*[title/abstract/keyword] or malignanc* [title/abstract/keyword] or cancer*[title/abstract/keyword]  #4 anorexias[title/abstract/keyword] or cachexia[title] or anorexia-cachexia syndrome[title]  #5 #1 or #3  #6 #2 or #4  #7 #5 and #6 |
| CINAHL | #1 neoplasm*[subject] or neoplasia*[subject] or tumor*[subject] or malignanc*[subject] or cancer*[subject]  #2 anorexia*[subject] or cachexia[title] or anorexia-cachexia syndrome[title]  #3 review[publication type] or meta-analysis[publication type] or systematic review*[subject]  #4 #1 and #2 and #3 |
| JBI | anorexia*[keyword] or cachexia[keyword] or anorexia-cachexia syndrome[keyword] |

| **Database** | **Search strategy** |
| --- | --- |
| CBM | #1 neoplasms[mesh]  #2 anorexia[mesh]  #3 review[mesh]  #4 meta analysis[mesh]  #5 cancer[title/abstract/keyword] or tumor[title/abstract/keyword] or malignanc*[title/abstract/keyword]  #6 anorexias[title/abstract/keyword] or cachexia[title/abstract/keyword] or anorexia-cachexia syndrome [title/abstract/keyword]  #7 systematic review*[title/abstract/keyword]  #8 #1 or #5  #9 #2 or #6  #10 #3 or #4 or #7  #11 #8 and #9 and #10 |
| CNKI | #1 neoplasms[title/abstract/keyword] or neoplasm[title/abstract/keyword] or tumor[title/abstract/keyword]  or cancer[title/abstract/keyword] or malignanc*[title/abstract/keyword]  #2 anorexias[title/abstract/keyword] or cachexia[title/abstract/keyword] or anorexia-cachexia syndrome [title/abstract/keyword]  #3 review[title/abstract/keyword] or systematic review*[title/abstract/keyword] or meta[title/abstract/keyword]  #4 #1 and #2 and #3 |
| WanFang | (neoplasms[title/abstract/keyword] or neoplasm[title/abstract/keyword] or tumor[title/abstract/keyword]  or cancer[title/abstract/keyword] or malignanc*[title/abstract/keyword]) and (anorexias [title/abstract/keyword] or cachexia[title/abstract/keyword] or anorexia-cachexia syndrome [title/abstract/keyword]) and (review[title/abstract/keyword] or systematic review*[title/abstract/keyword] or meta[title/abstract/keyword]) |

Abbreviations: CNKI, China National Knowledge Infrastructure; CBM, Chinese Bio-medical Literature Database; WanFang, China WanFang Database.
